# Fed-Batch Synthesis of Poly(3-Hydroxybutyrate) and Poly(3-Hydroxybutyrate-*co*-4-Hydroxybutyrate) from Sucrose and 4-Hydroxybutyrate Precursors by *Burkholderia sacchari* Strain DSM 17165

**DOI:** 10.3390/bioengineering4020036

**Published:** 2017-04-20

**Authors:** Miguel Miranda de Sousa Dias, Martin Koller, Dario Puppi, Andrea Morelli, Federica Chiellini, Gerhart Braunegg

**Affiliations:** 1 Université Pierre et Marie Curie UPMC, Institut national de la santé et de la recherche médicale INSERM, Centre national de la recherche scientifique CNRS, Institut de la Vision, Sorbonne Universités, 17 rue Moreau, 75012 Paris 06, France; migueldias670@hotmail.com; 2 Institute of Chemistry, University of Graz, NAWI Graz, Heinrichstrasse 28/III, 8010 Graz, Austria; 3 ARENA—Association for Resource Efficient and Sustainable Technologies, Inffeldgasse 21b, 8010 Graz, Austria; g.braunegg@tugraz.at; 4 BIOLab Research Group, Department of Chemistry & Industrial Chemistry, University of Pisa, UdR INSTM Pisa, Via Moruzzi, 13, 56124 Pisa, Italy; d.puppi@dcci.unipi.it (D.P.); a.morelli@dcci.unipi.it (A.M.); federica.chiellini@unipi.it (F.C.)

**Keywords:** 4-hydroxybutyrate, biopolymers, *Burkholderia sacchari*, copolyester, poly(3-hydroxybutyrate-*co*-4-hydroxybutyrate), polyhydroxyalkanoate (PHA), saccharose, sucrose, sugarcane

## Abstract

Based on direct sucrose conversion, the bacterium *Burkholderia sacchari* is an excellent producer of the microbial homopolyester poly(3-hydroxybutyrate) (PHB). Restrictions of the strain’s wild type in metabolizing structurally related 3-hydroxyvalerate (3HV) precursors towards 3HV-containing polyhydroxyalkanoate (PHA) copolyester calls for alternatives. We demonstrate the highly productive biosynthesis of PHA copolyesters consisting of 3-hydroxybuytrate (3HB) and 4-hydroxybutyrate (4HB) monomers. Controlled bioreactor cultivations were carried out using saccharose from the Brazilian sugarcane industry as the main carbon source, with and without co-feeding with the 4HB-related precursor γ-butyrolactone (GBL). Without GBL co-feeding, the homopolyester PHB was produced at a volumetric productivity of 1.29 g/(L·h), a mass fraction of 0.52 g PHB per g biomass, and a final PHB concentration of 36.5 g/L; the maximum specific growth rate *µ_max_* amounted to 0.15 1/h. Adding GBL, we obtained 3HB and 4HB monomers in the polyester at a volumetric productivity of 1.87 g/(L·h), a mass fraction of 0.72 g PHA per g biomass, a final PHA concentration of 53.7 g/L, and a *µ_max_* of 0.18 1/h. Thermoanalysis revealed improved material properties of the second polyester in terms of reduced melting temperature *T_m_* (161 °C vs. 178 °C) and decreased degree of crystallinity *X_c_* (24% vs. 71%), indicating its enhanced suitability for polymer processing.

## 1. Introduction

Polyhydroxyalkanoates (PHA) are a versatile group of microbial biopolyesters with properties mimicking those of petrol-based plastics. A growing number of described bacterial and archaeal prokaryotic species accumulate PHA as refractive granular inclusion bodies in the cell’s cytoplasm. PHA granules are surrounded by a complex membrane of proteins and lipids; these functional “carbonosomes” are typically accumulated under conditions of an excess exogenous carbon source in parallel with the limitation of a growth-essential component like the nitrogen source or phosphate [1,2,3,4]. Playing a major biological role, the presence of intracellular PHA supports bacterial survival under the conditions of carbon starvation. Moreover, PHA has pivotal functions in protecting cells against environmental stress conditions such as extreme temperature [5,6], exposure to oxidants [5,7], organic solvents [7], and UV-irradiation [6]. Depending on their composition, we distinguish homopolyesters, consisting of only one type of monomer, from heteropolyesters, composed of two or more types of monomers differing in their side chains (copolyesters) or both in their side chains and backbones (terpolyesters). In this context, the best known member of the PHA family, namely the homopolyester poly(3-hydroxybutyrate) (PHB), has restricted processability due to its high brittleness and crystallinity if compared to heteropolyesters consisting of different monomers such as 3-hydroxybutyrate (3HB), 3-hydroxyvalerate (3HV), 4-hydroxybutyrate (4HB), or 3-hydroxyhexanoate (3HHx) [8]. Changing PHA’s composition on the monomeric level offers the possibility to fine-tune the polymer properties (melting temperature *T_m_*, glass transition temperature *T_g_*, degree of crystallinity *X_c_*, degradability, elongation at break, or tensile strength) according to the customer’s demands [9]. Apart from utilization in its crude form, PHA can be processed together with compatible organic or inorganic materials to make various composites and blends with tailored properties in terms of density, permeability, tensile strength, (bio)degradability, crystallinity, etc. [10,11,12]. To an increasing extent, the processing of PHA with nanoparticles is reported to generate novel designer bio-plastics especially useful for, *inter alia*, “smart packaging” [13,14].

Nowadays, there is an emerging trend of substituting petrol-based plastics with sustainable “bio-alternatives” with low environmental impact, that are biodegradable and bio-based in their nature [15,16]. Nevertheless, PHA production is still challenged by cost-decisive factors which make them considerably more expensive than their petrochemical counterparts; in order to optimize PHA production economically, all single process steps have to be taken into account [4,17]. Enhanced downstream processing to recover intracellular PHA from the biomass [18,19,20,21], bioreactor design and process regime [22,23,24,25], and in-depth understanding of the kinetics of the bioprocess [26] are crucial factors when designing a new PHA production process. Nevertheless, the selection of the most suitable carbonaceous raw materials to be used as feedstocks for PHA biosynthesis is the issue that is most difficult to solve. In this context, there is an increasing trend towards the application of carbon-rich (agro) industrial waste materials to produce the so called “2nd generation PHA” [4]. Among these materials, the current literature familiarizes us with PHA production based on surplus whey [27], abundant lignocelluloses [28,29,30], waste lipids from animal processing [31,32,33], used plant-and cooking oils [34,35,36], crude glycerol from biodiesel production [37,38,39,40], plant root hydrolysates [30], extracts and hydrolysates of spent coffee ground [41,42], and molasses [43]. Such waste materials already performed well as substrates on the laboratory scale, but are still awaiting their implementation in industrial-scale PHA production processes. This is mainly due to problems associated with upstream processing, insecure supply chains, presence of inhibitory compounds, or fluctuating composition of the industrial waste streams [4]. An emerging trend in using industrial waste streams is recognized in the direct conversion of CO_2_ from industrial effluent gases [44]; here, cyanobacteria [45,46] or “Knallgasbacteria” [47] are potential cellular factories used to convert CO_2_ to “3rd generation PHA” and additional valued products. Although also promising on the laboratory scale, development of these processes to industrial maturity has hitherto not been reached [44,45,46,47].

Apart from 2nd and 3rd generation PHA, the production of PHA based on materials relevant for food and feed purposes (“1st generation PHA”) can also become economically viable given the integration of PHA-production facilities into existing production lines, where the raw material is generated [48]. This is successfully demonstrated at PHB Industrial SA (PHBISA), a company located in the Brazilian state of São Paolo. PHBISA is involved in the cane sugar business, predominantly fermenting hydrolyzed sucrose to bioethanol, and selling sucrose in its native form; a small part of sucrose is currently converted to PHA in a pilot plant with 100 ton annual capacity, and marketed under the trade mark Biocycle^TM^ [48]. Remarkably, this bio-refinery process works energetically autarkic by the thermal conversion of surplus sugarcane bagasse to generate steam and electrical energy, which are used in the bioprocesses and the distillation for ethanol recovery. Moreover, distillative ethanol recovery generates a mixture of medium-chain-length alcohols (butanol, pentanol, etc.), which are used by the company for extractive PHA recovery from microbial biomass. This strategy saves expenses for the typically applied and often halogenated extraction solvents, which considerably contribute to the entire PHA production costs [48]. Currently, PHA production at PHBISA is carried out using the well-known production strain *Cupriavidus necator*, a eubacterial organism lacking the enzymatic activity for sucrose cleavage; hence, sucrose hydrolysis of the monomeric sugars (glucose and fructose) is a needed laborious operation step during upstream processing. For further optimization of this sucrose-based PHA production process, the assessment of alternative production strains appears reasonable. Such new whole-cell biocatalysts should fulfill some requirements: Growth rate and volumetric PHA productivity that are competitive with the data known for *C. necator*; direct sucrose conversion without the need for hydrolysis; temperature optima in the slightly thermophile range (in order to save cooling costs, a decisive cost factor under the climatic conditions prevailing in São Paolo); and last but not least, the strain should be able to produce copolyesters with advanced material properties.

A strain that appears promising in all these criteria is *Burkholderia sacchari* IPT 101 (DSM 17165), originally isolated from the soil of Brazilian sugarcane fields and investigated by Brämer and colleagues [49]. The strain is reported to accumulate high amounts of PHA inter alia from glucose [39,50], sucrose [49,50], glycerol [39,50], organic acids [51], pentose-rich substrate cocktails mimicking hydrolysates of bagasse [52], and hydrolyzed straw [53]. Aimed at the optimized utilization of lignocellulose hydrolysate, efforts are currently devoted to further improve the strain’s substrate conversion ability in terms of xylose uptake [54]. PHA production by this organism and its mutant strains was demonstrated both in mechanically stirred tank bioreactors [52,53,55,56] and in airlift bioreactors [57]. As a drawback, the wild type strain displays insufficient ability for 3HV formation from structurally related precursors such as propionic acid, which is in contrast to pronounced 3HV formation by its mutant strain *B. sacchari* IPT 189 [54,56,58,59]. Formation of copolyesters consisting of 3HB and 4HB, hence P(3HB-*co*-4HB), was successfully demonstrated by co-feeding glucose or wheat straw hydrolysate (WSH) and the 4HB-related precursor compound γ-butyrolactone (GBL) [49]. Only recently has the production of copolyesters of 3HB and 3-hydroxyhexanoate (3HHx) by genetically engineered *B. sacchari* been reported [60]. In the present study, we demonstrate for the first time the feasibility of high-cell density production of PHB and P(3HB-*co*-4HB) by *B. sacchari* based on saccharose from PHBISA and the 4HB-precursor GBL, and for the first time, GBL’s saponified form, 4-hydroxybutyrate sodium salt (Na-4HB). Furthermore, by addressing the contradictory literature information on the optimum temperature at which this organism thrives [50,51,52,53,54], we adapted the strain to an elevated cultivation temperature of 37 °C according to the requirements at the Brazilian production site [48,61]. Detailed kinetic data under controlled conditions in laboratory bioreactors, and an in-depth comparison of the polymer data of PHB and P(3HB-*co*-4HB), respectively, are provided.

## 2. Materials and Methods 

### 2.1. Strain Maintenance and Adaptation to Elevated Temperature

*Burkholderia sacchari* DSM 17165 was purchased from DSMZ, Germany, and were grown on solid media plates (medium according to Küng [62] with 10 g/L of sucrose as the carbon source and 2 g/L ammonium sulfate as the nitrogen source). In two-week intervals, single colonies were transferred to new plates and incubated at 37 °C. All mineral components of the medium were purchased in p.a. quality (Company Roth, Graz, Austria), whereas sugarcane sucrose was obtained as unrefined saccharose directly from PHBISA.

### 2.2. Shaking Flask Cultivation to Assess Production of 4HB-Containing PHA

For preparation of pre-cultures, fresh single colonies from solid media were transferred to 100 mL of a liquid mineral medium containing the following components (g/L): KH_2_PO_4_, 9.0; Na_2_HPO_4_·2H_2_O, 3.0; (NH_4_)_2_SO_4_, 2.0; MgSO_4_·7H_2_O, 0.2 g; CaCl_2_·2H_2_O, 0.02; NH_4_Fe(III)citrate, 0.03; SL6, 1.0 (mL/L); sucrose, 15.0. These pre-cultures were incubated at 37 °C under continuous shaking; after 24 h, 5 mL of these pre-cultures were used for inoculation of four flasks each containing 100 mL of the minimal medium. The pH-value was adjusted to 7.0. After 8 h of incubation at 37 °C, 4HB-precursors were added to the cultures as follows: Two of the flasks were supplied with a solution of GBL, and two cultures with a solution of Na-4HB. Both solutions were added in a quantity to achieve a final precursor (GBL or the anion of 4HB, respectively) concentration of 1.5 g/L each. 15 h later, the re-feed of 4HB precursors was accomplished using the same quantity (1.5 g/L). After 47 h of cultivation, the experiment was stopped and the fermentation broth was analyzed for cell dry mass (CDM), PHA mass fraction in CDM, and PHA composition (fractions of 3HB and 4HB) (analytical methods *vide infra*).

### 2.3. Bioreactor Cultivations

#### 2.3.1. PHB Production

Single colonies of *B. sacchari* were used to inoculate 100 mL (pre-cultures) of the medium according to Küng as described above. These pre-cultures were incubated (37 °C) for 36 h; then, 5 mL each of these pre-cultures were used for the inoculation of seven shaking flasks each containing 250 mL of the minimal medium. These cultures were incubated under continuous shaking at 37 °C for 36 h, until high cell densities (8–9 g/L) were reached, and two of them were used to inoculate a Labfors 3 bioreactor (Infors, CH) with an initial working volume of 1.5 L (1.0 L fresh medium with compounds calculated for 1.5 L plus 0.5 L inoculum). At the start of the cultivation, sucrose and (NH_4_)_2_SO_4_ amounted to 15 g/L and 2.5 g/L, respectively. The set point for dissolved oxygen concentration (DOC) was 40% of the air saturation during the growth phase, and 20% during nitrogen-limited conditions; DOC was controlled by automatic adjustment of the stirrer speed and aeration rate. The pH-value was set to 7.0 and controlled automatically by the addition of H_2_SO_4_ (10%) to decrease the pH-value, and ammonia solution (25%) during the growth phase or NaOH (10%) during the accumulation phase to increase the pH-value. Hence, during the growth phase, the addition of the nitrogen source was coupled with pH-value correction. The cultivation was carried out at 37 °C. The time points of sugar addition (50% w/w aqueous solution of Brazilian sugarcane saccharose) are indicated in Figure 2 by arrows; the total amount of sucrose solution refeed amounted to 360 g.

#### 2.3.2. P(3HB-*co*-4HB) Production:

This process was based on inoculum preparation according to the previous experiment. Cultivation in the bioreactor was performed using a minimal medium identical to the process at the company PHBISA (g/L): KH_2_PO_4_, 5.0; (NH_4_)_2_SO_4_, 2.5; MgSO_4_·7H_2_O, 0.8; NaCl; 1.0; CaCl_2_·2H_2_O, 0.02; NH_4_Fe(III)citrate, 0.05; trace element solution SL6 2.5 mL/L; sucrose 30; and the 4HB-precursor 4HB was provided by dropwise addition during the accumulation phase (total addition of GBL 15.5 g/L). Also in this case, a Labfors 3 bioreactor with an initial working volume of 1.5 L (1.0 L fresh medium with compounds calculated for 1.5 L plus 0.5 L inoculum) was used with the same basic parameters (DOC, T, pH-value) as described for the previous fermentation. The time points of sugar addition are indicated in Figure 7 by the arrows; the total amount of sucrose refeed amounted to 207 g of solution.

### 2.4. Cell Dry Mass (CDM) Determination

A gravimetric method was used to determine CDM in the fermentation samples. Five mL of the culture broth was centrifuged in pre-weighed glass screw-cap tubes for 10 min at 10 °C and 4000 rpm in a Heraeus Megafuge 1.0 R refrigerated centrifuge (Heraeus, Hanau, Germany). The supernatant was decanted, and subsequently used for substrate analysis. The cell pellets were washed with distilled water, re-centrifuged, frozen, and lyophilized (freeze-dryer Christ Alpha 1-4 B, Martin Christ Gefriertrocknungsanlagen GmbH, Osterode am Harz, Germany) to constant mass. CDM was expressed as the mass difference between the tubes containing cell pellets minus the mass of the empty tubes. The determination was done in duplicate. The lyophilized pellets were subsequently used for determination of intracellular PHA as described in the next paragraph.

### 2.5. Analysis of PHA Content in Biomass and Monomeric PHA Composition

For the analysis of PHA, standards of P(3HB-*co*-5.0%-3HV) (Biopol^TM^, ICI, London, UK) were used for determination of the 3HB content; for determination of 4HB, “self-made” Na-4HB (next paragraph) was used as the reference material. Intracellular PHA in lyophilized biomass samples was transesterificated to volatile methyl esters of hydroxylkanoic acids via Braunegg’s acidic methanolysis method [63]. Analyses were carried out with an Agilent Technologies 6850 gas chromatograph (30-m HP5 column, Hewlett-Packard, Palo Alto, CA, USA; Agilent 6850 Series Autosampler). The compounds were detected by a flame ionization detector; the split ratio was 1:10.

### 2.6. Preparation of Na-4HB

Na-4HB was synthesized by manually dropping a defined quantity of GBL into an equimolar aqueous solution of NaOH under continuous stirring and cooling. The obtained solution of Na-4HB was further frozen and lyophilized (freeze-dryer Christ Alpha 1–4 B) to obtain Na-4HB as a white powder. This powder was applied as a reference material for the analysis and as a co-substrate.

### 2.7. Substrate Analysis

The determination of carbon sources (sucrose and its hydrolysis products glucose, fructose, Na-4HB, and GBL) was accomplished by HPLC-RI using an Aminex HPX 87H column (thermostated at 75 °C, Biorad, Hercules, CA, USA), a LC-20AD pump, a SIC-20AC autosampler, a RID-10A refractive index detector, and a CTO-20AC column oven. Pure sucrose, glucose, fructose, Na-4HB, and GBL were used as standards for external calibration. Isocratic elution was carried out with 0.005 M H_2_SO_4_ at a flow rate of 0.6 mL/min.

### 2.8. Analysis of Nitrogen Source (NH_4_^+^)

The determination of the nitrogen source was done using an ammonium electrode (Orion) with ammonium sulfate solution standards (300–3000 ppm) as described previously [39].

### 2.9. PHA Recovery

After the end of the experiments, the fermentation broth was *in situ* pasteurized (80 °C, 30 min). Afterwards, the biomass was separated from the liquid supernatant via centrifugation (12,000 g; Sorvall^®^ RC-5B Refrigerated Superspeed centrifuge, DuPont Instruments, Wilmington, NC, USA), frozen, and lyophilized (freeze-dryer Christ Alpha 1-4 B). Dry biomass was decreased by overnight stirring with a 10-fold mass of ethanol; after drying, PHA was extracted from the degreased, dried biomass by continuous overnight stirring in a 25-fold mass of chloroform in light-protected glass vessels. The solution containing the PHA was separated by vacuum-assisted filtration, and concentrated by evaporation of the major part of the solvent (Büchi Rotavapor^®^ R-300). This concentrated PHA solution was dropped into permanently stirred ice-cooled ethanol. Precipitated PHA filaments of high purity were obtained by vacuum-assisted filtration, dried, and subjected to polymer characterization (*vide infra*).

### 2.10. Polymer Characterization

#### 2.10.1. Molecular Mass Distribution

Gel Permeation Chromatography (GPC) analysis was carried out on a Waters 600 model (Waters Corporation, Milford, MA, USA) equipped with a Waters 410 Differential Refractometer and two PLgel 5 µm mixed-C columns (7.8 × 300 mm^2^). The mobile phase constituted by chloroform (CHROMASOLV^®^ for HPLC amylene stabilized, Sigma-Aldrich, Milan, Italy) was eluted at a flow rate of 1 mL/min. Monodisperse polystyrene standards were used for calibration (range 500–1.800,000 g/mol). Samples were prepared at a concentration of ca. 0.5% (*w*/*v*).

#### 2.10.2. Thermoanalysis

Differential Scanning Calorimetry (DSC) analysis was performed using a Mettler DSC-822E instrument (Mettler Toledo, Novate Milanese, Italy) under a nitrogen flow rate of 80 mL/min. The analysis was carried out in the range from −20 to 200 °C at a heating and cooling rate of 10 °C/min. By considering the second heating cycles in the thermograms, the glass transition temperature (*T_g_*) was evaluated by analyzing the inflection point, while the melting temperature (*T_m_*) and crystallinity (*X_c_*) was evaluated by analyzing the endothermic peak. *X_c_* was determined by considering the value of the melting enthalpy of 146 J/g for the 100% crystalline PHB. Both characterization tests were carried out on five replicates for each kind of sample and the data were presented as mean ± standard deviation. Statistical differences were analyzed using one-way analysis of variance (ANOVA), and a Tukey test was used for post hoc analysis. A *p*-value < 0.05 was considered statistically significant.

## 3. Results

### 3.1. Impact of 4HB-Precursors GBL and Na-4HB on Poly-(3-hydroxybutyrate-co-4-hydroxybutyrate) (P(3HB-co-4HB)) Biosynthesis by Burkholderia sacchari DSM 17165 on Sucrose

**Figure 1:**
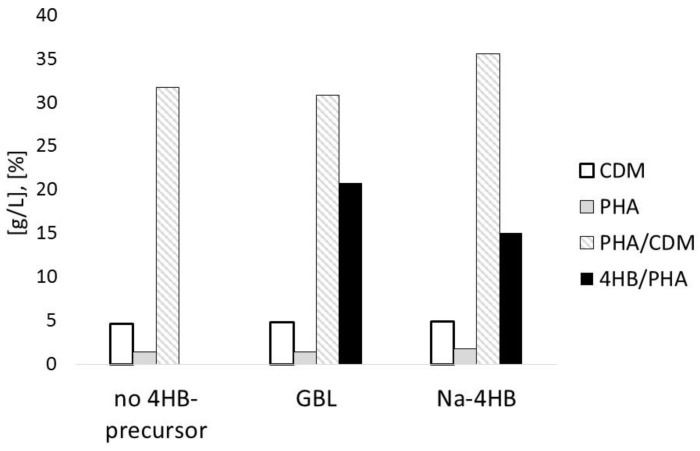
Cell dry mass (CDM) (g/L), polyhydroxyalkanoate (PHA) (g/L), mass fraction of PHA in CDM (%), and mass fraction of 4-hydroxybutyrate (4HB) in PHA (%): *B. sacchari* after 47 h of cultivation on 15 g/L sucrose and 4HB-precursors γ-butyrolactone (GBL) or Na-4HB (precursor addition: 1.5 g/L after 8 h, refeed of 1.5 g/L after 15 h).

Figure 1 illustrates the outcomes of the shaking flask experiment comparing the effect of adding 4HB-precursors GBL and Na-4HB to *B. sacchari* cultivated on sucrose as main carbon source. After 47 h of incubation, the CDM concentration was in the range of 5 g/L in all experimental setups. Final PHA concentrations amounted to 1–2 g/L without significant differences between the individual cultivation setups. Using GBL as the 4HB-related precursor, PHA fractions in the CDM were slightly lower than in the case of using Na-4HB, but almost identical to the setups without precursor addition (ca. 30% vs. ca. 35%, respectively). The 4HB fractions in PHA (4HB/PHA) differ in dependence on the applied precursor; using GBL, this value amounted to 20.8%, while it was only 14.1% when using Na-4HB. As expected, the setups cultivated on sucrose as the sole carbon source (no addition of 4HB-related precursors) resulted in the generation of the PHB homopolyester. Here, it has to be emphasized that it is not clear from the available data if the generated polyester is definitely a P(3HB-*co*-4HB) copolyester with random distribution of the individual building blocks, a blend of homopolymers consisting of 3HB or 4HB, respectively, or a blend of different P(3HB-*co*-4HB) copolyesters with different 4HB fractions.

### 3.2. Poly(3-hydroxybutyrate) (PHB) Production with Burkholderia sacchari on the Bioreactor Scale; Sucrose as the Sole Carbon Source

#### 3.2.1. Bioprocess

This experiment aimed to test a medium similar to the one used at the industrial company PHBISA for sucrose-based PHA production by *C. necator*, and to study its influence on the kinetic data and on the polymer production (*cf.* Materials and Methods section). Of major importance, it was intended to considerably increase the concentration of the residual biomass and to achieve higher productivities for PHA. This was accomplished using an advanced strategy for adding the nitrogen source (NH_4_^+^) during the microbial growth phase by coupling the addition of the nitrogen source with the correction of the pH-value. Instead of a periodic re-feed of (NH_4_)_2_SO_4_ solution to maintain the nitrogen concentration at the desired level, NH_4_OH was used as a base for correction of the pH-value and, at the same time, to provide the nitrogen needed by the strain to grow. Hence, the addition of the nitrogen source was directly coupled to the excretion of acidic metabolites during the growth phase. After 12.5 h of fermentation, the NH_4_OH solution as the pH-correction agent was replaced by NaOH solution (20%) in order to provoke a nutritional stress by limitation of the nitrogen source to stop the biomass formation and to enhance PHA production; this time point is marked by a full line in Figure 3. The depletion of the nitrogen source occurred after 19 h of cultivation.

**Figure 2:**
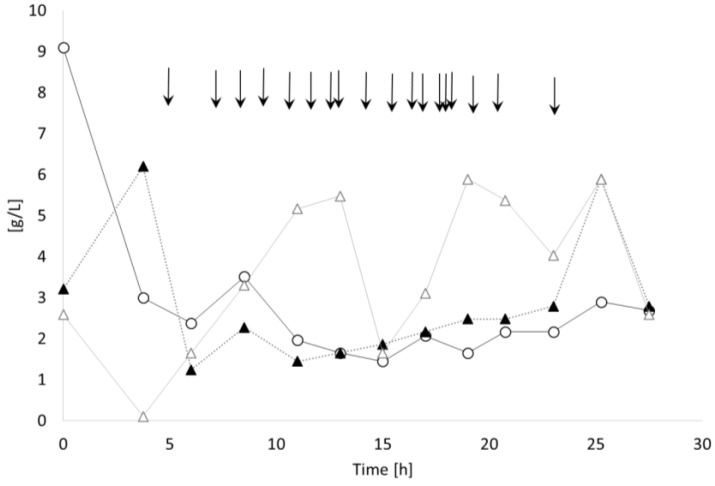
Substrate time curves: *B. sacchari* on sucrose without supplementation of 4HB-precursors. Open spheres: sucrose; black triangles: glucose; open triangles: fructose. Arrows indicate the time points of pulse feedings of the sucrose solution.

Figure 2 illustrates the time curves of the sugar concentrations (sucrose, glucose, and fructose). It is easily seen that the strain possesses the metabolic ability to rapidly hydrolyze the disaccharide sucrose to its monomeric sugars by the excretion of an extracellular invertase enzyme. Immediately after inoculation, hydrolysis started, resulting in about 9 g/L sucrose and 6 g/L of monomers (glucose plus fructose) already present in the first sample taken at *t* = 0 h. The time points of sucrose additions are marked by arrows in Figure 2. Remarkably, the concentrations of the two monosaccharides do not follow the same trend with time, which might be due to the changing conversion rates of the individual monomers (glucose or fructose, respectively) with the changing environmental (nutritional) conditions during the cultivation. Mathematical modelling of the data to elucidate the metabolic processes should therefore be performed in follow-up experiments by specialists in the field of metabolic flux analysis. A total quantity of 360 g sucrose solution was added during the process. A total sugar consumption of 29.14 g/(L·h) was observed, and a conversion yield of sugar to CDM of 0.18 g/g (calculated for the entire sugar addition and also encompassing the not utilized sugar in the spent fermentation broth) (Table 1). Limitation of the carbon source was avoided during the entire cultivation period by permanent monitoring (HPLC) and re-feeding (Figure 2).

**Figure 3:**
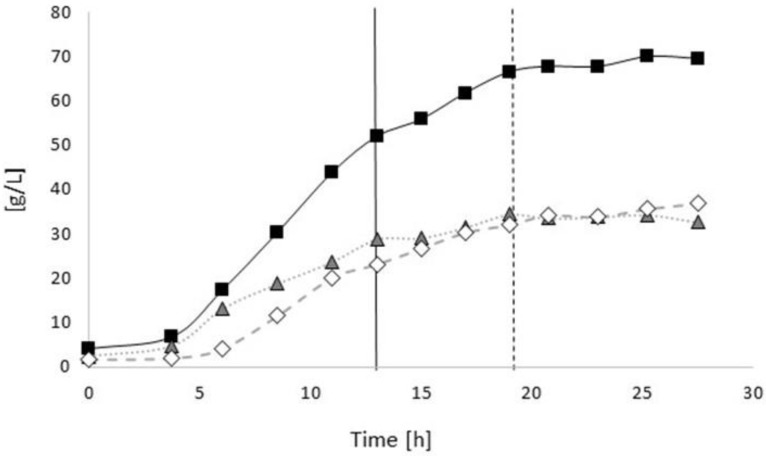
Time curves of CDM, residual biomass, and PHB concentration: *B. sacchari* on sucrose without supplementation of 4HB-precursors. Black squares: CDM; open rhombi: PHA; grey triangles: residual biomass. Full black line: Exchange of NH_4_OH solution by NaOH solution as the pH-corrective agent, dashed line: start of nitrogen depletion in the medium.

Figure 3 illustrates the time curves of the CDM, residual biomass, and PHA during the process. After the onset of nitrogen limitation after 19 h (indicated by a dashed line in Figure 3), the concentration of the residual biomass remained constant (35 g/L), whereas the PHA concentration increased, reaching a maximum concentration of 36.5 g/L at the end of the fermentation. This corresponds to a final CDM concentration of 70 g/L. Due to the fact that no 4HB-related precursors were supplied, homopolyester PHB was accumulated. The volumetric productivity for PHB, calculated for the entire process, amounted to 1.29 g/(L·h). For the entire process (*t* = 0 to 27.5 h), the yield for the conversion of sugars to CDM amounted to 0.18 g/g, whereas during the nitrogen-limited phase of cultivation, a conversion yield for sugars to PHB of 0.08 g/g was evidenced (Table 1).

**Figure 4:**
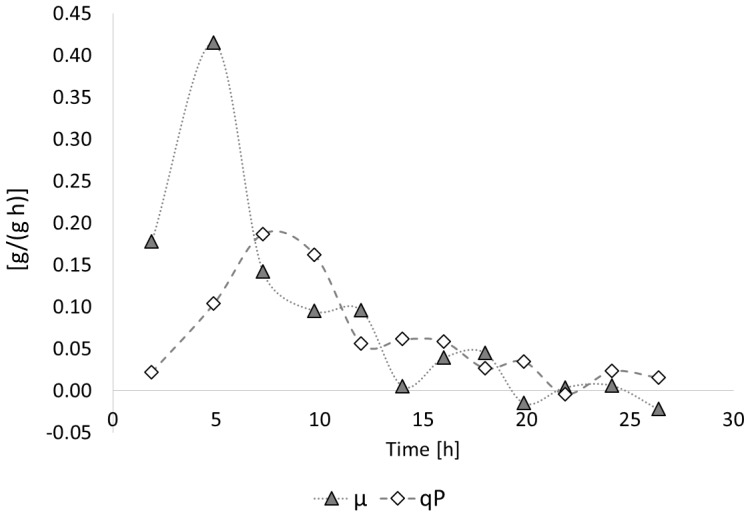
Time course of the specific growth rate *µ* and specific PHA production rate *q_P_*: *B. sacchari* on sucrose without supplementation of 4HB-precursors. Full black line: Exchange of NH_4_OH solution by NaOH solution as the pH-corrective agent; dashed line: start of nitrogen depletion in the medium.

Figure 4 illustrates the time curves of the specific growth rate *µ* and the specific product (PHB) formation rate *q_P_* for the entire process. Here, it is visible that the maximum specific growth (*µ_max_* = 0.41 1/h) was monitored at around 5 h of cultivation. For the entire growth phase (*t* = 3.75–13 h), *µ_max_* was determined with 0.15 1/h by plotting the natural logarithm LN of the residual biomass concentration vs. time. After the exchange of NH_4_OH by the NaOH solution and the resulting depletion of the nitrogen source, the specific growth tremendously decreased, and a slight decrease of the residual biomass concentration, indicated by the negative values for *µ* in Figure 4, was observed. The highest specific PHB production was observed starting from the onset of the exponential growth phase (*t* = 5 h) until the start of nitrogen depletion at *t* = 12 h; a *q_P_* of about 0.19 g/(g·h) was measured for the period between the two subsequent samplings at *t* = 6 and 8.5 h. In later periods of the process, only a slight increase of PHB production, manifested by low values for *q_P_*, was observed.

#### 3.2.2. Polymer Characterization:

After the end of the experiment, the biomass was separated from the liquid supernatant via centrifugation, and was frozen and lyophilized. The dry biomass was decreased with ethanol and the polymer was extracted using chloroform. The weight average molecular mass (Mw) and the polydispersity (*P_i_*; Mw/Mn) values of the extracted homopolymer were determined by gel permeation chromatography (GPC). The Mw was 627 ± 13 kDa and *P_i_* was 2.66 ± 0.13 kDa (Table 1). Differential scanning calorimetry (DSC) analysis was carried out to determine the glass transition temperature (*T_g_*), melting temperature (*T_m_*), and crystallinity (*X_c_*) of the PHB samples. Analysis of the obtained data showed that the *T_g_* of the produced PHB was 1.0 ± 0.6 °C and the *T_m_* was 177.6 ± 0.6 °C, while *X_c_* was 70.9% ± 0.9%.

### 3.3. Controlled Poly(3-Hydroxybutyrate-co-4-Hydroxybutyrate) (P(3HB-co-4HB)) Production with Burkholderia sacchari on the Bioreactor Scale: Sucrose plus GBL as Carbon Subsubstrates.

#### 3.3.1. Bioprocess

Based on the results from the shaking flask scale reported in this study and previous findings which confirmed *B. sacchari*’s potential to produce PHA containing 4HB by co-feeding sucrose and 4HB-related precursor compounds, this material was produced under controlled conditions at the bioreactor scale. It was aimed at generating a residual biomass concentration of about 20 g/L and a PHA mass fraction in CDM exceeding 60 g/L in order to be competitive with the *C. necator*—mediated sucrose-based PHA production process at PHBISA.

**Figure 5:**
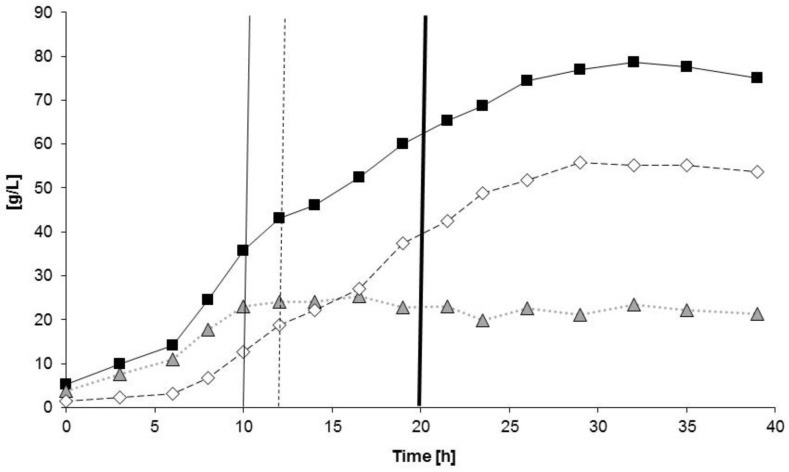
Time curves of product concentrations: *B. sacchari* on sucrose and the addition of γ-butyrolactone (GBL) as 4HB precursor. Black squares: CDM; open rhombi: PHA; grey triangles: residual biomass. Thin black line: Exchange of NH_4_OH solution by NaOH solution as the pH-corrective agent; dash line: start of nitrogen depletion in the medium; bold black line: start of GBL feed.

**Figure 6:**
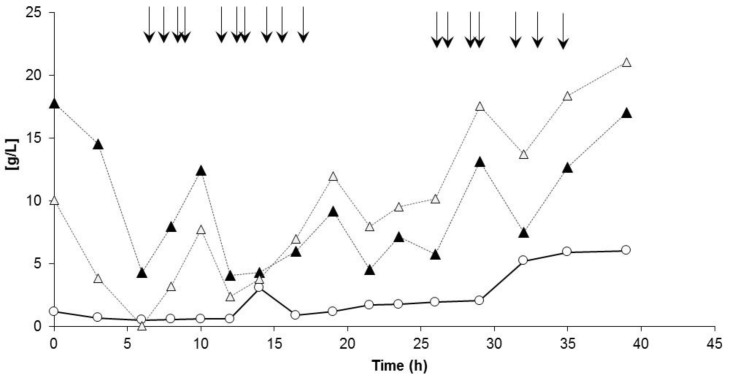
Actual concentrations of sugars: *B. sacchari* on sucrose and the addition of GBL as 4HB precursor. Arrows indicate the refeed with sucrose solution. Open spheres: sucrose; black triangles: glucose; open triangles: fructose.

**Figure 7:**
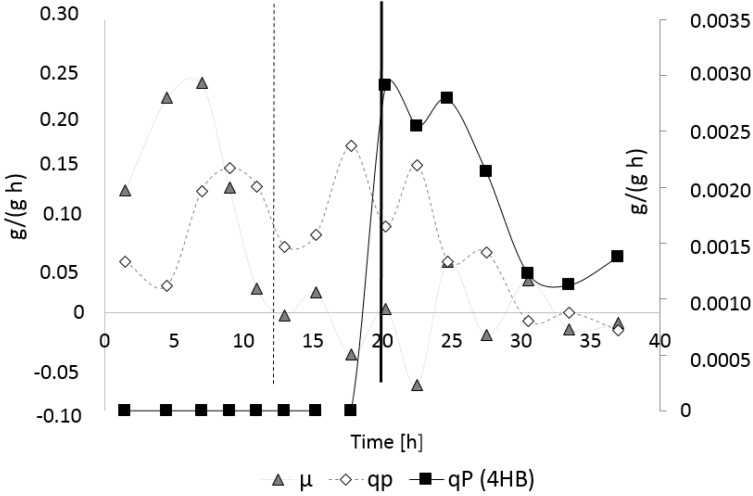
Time course of the specific growth rate *µ* and specific PHA production rate *q_P_* (left axis): *B. sacchari* on sucrose and the addition of GBL as 4HB precursor. Thin black line: Exchange of NH_4_OH solution by NaOH solution as the pH-corrective agent; dashed line: start of nitrogen depletion in the medium; bold black line: start of GBL feed.

Figure 5 shows the time curves of the CDM, PHA, and residual biomass, whereas Figure 6 illustrates the corresponding time curves of the sugar concentrations; again, arrows mark the time points of sucrose addition. Also in this cultivation, the nitrogen source (NH_4_^+^) served as the growth-limiting factor. NH_4_^+^ was added continuously during the growth phase as aqueous NH_4_OH solution (25%) according to the response of the pH-electrode. The maximum specific growth rate *µ_max_* measured between two subsequent samplings (*t* = 6–8 h) amounted to 0.23 1/h for the entire growth phase (*t* = 0–10 h), and the *µ_max_* for the entire exponential growth phase was determined to be 0.18 1/h. About 21 g/L of catalytically active residual biomass was produced until the onset of nitrogen depletion. Figure 7 shows the time curve of the main carbon source sucrose and its hydrolysis products glucose and fructose, which are produced by the extracellular invertase excreted by the organism; again, the rapid hydrolysis of sucrose is evident.

After 10 h of fermentation, the nitrogen source supply was stopped by exchanging NH_4_OH with NaOH as the pH-value correction agent; now, the second phase of the process was initiated (accumulation phase). During this phase, the time curve of the residual biomass was constant and the increase of CDM until the end of the experiment was only due to the increasing intracellular concentration of PHA (see Figure 5). It is visible that already during the exponential phase of the microbial growth (*t* = 7–10 h) that considerable amounts of PHA were produced (“growth associated product formation”). During the phase of product formation, GBL was added dropwise in order to not move into inhibiting concentration ranges. The actual GBL concentration was always below the detection limit when analyzing the samples; hence, GBL was completely converted by the cells. During the process, a total of 15.5 g/L GBL was added to the culture, distributed to a total of ten pulses of the substrate feed.

At the end of the process, the final concentrations of CDM and PHA of 75.1 g/L and 53.7 g/L, respectively, were achieved, corresponding to a PHA mass fraction in CDM of 71.5%. The total PHA concentration remained constant from *t* = 27.5 h. The volumetric productivity of PHA for the entire process and the conversion yield of sugar to CDM were calculated as 1.87 g/(L·h) and 0.38 g/g, respectively, which signifies an enormous enhancement in comparison to the previous experiment (Table 1).

Figure 7 illustrates the time curves of the specific growth rate *µ*, the specific PHA production rate *q_P_*, and the specific 4HB production rate for the entire process. Again, starting with nitrogen limitation at about *t* = 12 h, the values for *µ* drastically decreased, whereas the specific PHA productivity *q_P_* reached its highest values under nitrogen limited conditions; the maximum value for *q_P_* was reached between *t* = 16.5 and 19 h, and amounted to 0.17 g/(g·h). Maximum specific 4HB production occurred between *t* = 20 and 35 h, and was calculated with about 0.003 g/(g h).

**Figure 8:**
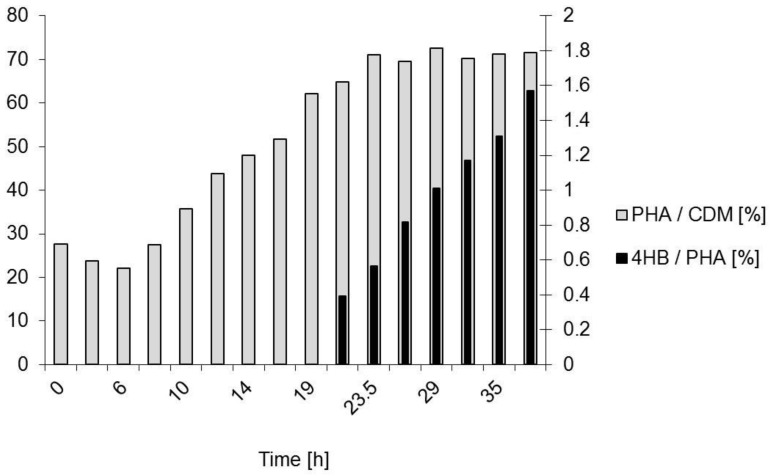
*B. sacchari* on sucrose and the addition of GBL as 4HB precursor. Composition of PHA during the process. Grey bars: Mass fraction of PHA in CDM (left axis). Black bars: Molar 4HB fraction on PHA (right axis). GBL addition started at *t* = 20 h.

Co-feeding of GBL started after 20 h; until this time, the PHB homopolyester was produced (Figure 7 and Figure 8). Starting with the sample taken at *t* = 23.5 h, 4HB-building blocks were detected in the polymer. The achieved 4HB fraction in PHA at the end of the fermentation was determined with 1.6% (mol/mol). The time curve of the polyester composition is illustrated in Figure 8. The essential process results are collected in Table 1 and directly compared with the outcomes of the previous process for the PHB production.

#### 3.3.2. Polymer Characterization:

After the end of the experiment, the biomass was separated from the liquid supernatant via centrifugation, and was frozen and lyophilized. The dry biomass was decreased with ethanol and the polymer was extracted using chloroform. The Mw and *P_i_* values of the extracted copolymer, determined by GPC, were 315 ± 24 kDa and 2.51 ± 0.15 kDa, respectively (Table 1). Statistical differences analyses showed that the Mw of P(3HB-*co*-4HB) was significantly lower than that of PHB. In addition, analysis of the DSC data showed that P(3HB-*co*-4HB) had significantly lower *X_c_* (24.0% ± 3.6%) and *T_m_* (160.9 ± 0.8 °C) than PHB, while *T_g_* was in the same range (1.8 ± 0.2 °C).

Table 1 compares both kinetic data and data from polymer characterization of both bioprocesses on the bioreactor scale.

## 4. Discussion

### 4.1. Bioprocess

The organism *B. sacchari* DSM 17165 possesses the desired ability to produce 4HB-containing PHA from sucrose plus both investigated 4HB precursors GBL and Na-4HB. The successful conversion of GBL towards 4HB building blocks is in agreement to previous findings reported by Cesário, who used glucose or WSH plus GBL for P(3HB-*co*-4HB) biosynthesis by this strain. These authors also tested P(3HB-*co*-4HB) production by this organism by using 1,4-butanediole as the 4HB-related precursor, revealing the incorporation of 4HB by GBL supplementation and the strain’s inability to utilize 1,4-butanediole. No reports were previously available on the utilization of Na-4HB by this strain. The results reported by Cesário et al. show varying PHA fractions in CDM for the fed-batch cultivation of *B. sacchari* on glucose/GBL mixtures, dependent on the ratio of glucose/GBL. Cultivation on pure glucose resulted in 49.2% PHB in CDM; this value decreased with increasing GBL portions in the feed stream to only 7.1% using GBL as the sole carbon source [28]. In our shaking flask setups, the rather modest precursor supplementation of 1.5 g/L neither significantly impacted the CDM production or the PHA fraction in CDM compared to the precursor-free setups (sucrose as the sole carbon source). Remarkably, the application of the GBLs saponified from Na-4HB resulted in considerably lower 4HB fractions in PHA than observed when using the annular lactone (GBL) (21% vs. 14%). As assumed for *C. necator* [64] and *Hydrogenophaga pseudoflava* [65], GBL is imported into the cells as an intact lactone ring, which is opened only intracellularly. According to Valentin et al., only a part of 4HB is converted to 4-hydroxybutyryl-CoA (4HB-CoA) in the cells, whereas 4HB’s major share is converted to succinic acid semialdehyde and succinic acid, which finally undergo conversion to the 3-hydroxybutyryl-CoA (3HB-CoA) precursor acetyl-CoA. PHA synthase in turn polymerizes 3HB-CoA and 4HB-CoA to P(3HB-*co*-4HB) [66].

As shown previously [39,52,53,55,57] and confirmed by the present work, nitrogen limitation is a suitable approach to boost PHA biosynthesis by *B. sacchari*. Generally, the strategy to constantly supply a nitrogen source by coupling the NH_4_OH supply to microbial growth by automatically responding to the signal of the pH-electrode was performed successfully to rapidly generate a high concentration of catalytically active biomass at a high specific growth rate. Only about 9 h (PHB production) or 12 h (production of 4HB-containing PHA) were needed to boost the concentration of the residual biomass above 20 g/L. This shows significant progress to comparable experiments carried out by Rocha and colleagues, who used the same strategy and achieved a maximum residual biomass of about 16 g/L after 24 h of cultivation using the mutant *B. sacchari* IPT 189 [55]. The maximum growth rates *µ_max_* obtained in our experiments (0.15 1/h for the first, 0.18 1/h for the second bioreactor cultivation; calculated for the entire growth phase; 0.41 and 0.23 1/h maximum valued between two subsequent samplings) can be compared to related reports found in the literature; for shaking flask cultivations of *B. sacchari* LFM 101 on sucrose, Nascimento et al. report a *µ_max_* of 0.544 and 0.546 1/h at 30 and 35 °C, respectively [50]. At the bioreactor scale, Rocha et al. obtained a *µ_max_* of 0.4 1/h for the first 10 hours of continuous cultivation of *B. sacchari* IPT 189 [55]; this value was also obtained by da Cruz Pradella with *B. sacchari* IPT 189 by using a fedbatch feeding regime in an airlift reactor [57]. Reliable *µ_max_* values from the bioreactor scale cultivations of our production strain *B. sacchari* IPT 101 (DSM 17165) are available for xylose-based experiments, where *µ_max_* amounted to 0.07–0.21 1/h with dependence on the initial xylose concentration [52]. Using glucose during the growth phase, Rodriguez-Contreras obtained a *µ_max_* of 0.42 1/h [39]. Testing the effect of GBL on the growth of *B. sacchari* in shaking flask setups, Cesário et al. noticed a continued decrease of *µ_max_* from 0.32 to 0.19 1/h with GBL concentrations increasing from 5 to 40 g/L, with 40 g/L glucose as the main carbon source. In this study, *µ_max_* was unfortunately not reported for the fedbatch cultivations in the bioreactors for the production of PHB and P(3HB-*co*-4HB) [53].

Furthermore, we demonstrated that the organism can successfully be cultivated at an elevated temperature of 37 °C, which is beneficial for large scale operation in reactors integrated into the production facilities of the Brazilian sugarcane industry [48,61]. The cultivation temperature of 37 °C is in contrast to previous literature reports for this organism and its close relatives. Generally, 30 °C is reported as the optimum temperature to efficiently thrive most *B. sacchari* sp. [50]. In a mechanically stirred tank bioreactor, Raposo and colleagues cultivated the same strain for the production of PHB, xylitol, and xylonic acid at a temperature of 32 °C [61], whereas 30–32 °C was used by da Cruz Pradella et al. to culture its mutant strain *B. sacchari* IPT 189 for PHB biosynthesis in an airlift reactor [57], or by Rocha and colleagues in continuously operated bioreactor cultivations [55]. *B. sacchari* LFM 101, a strain that is most likely closely related to our production strain, was only recently tested by Nascimento et al. for PHA production on sucrose, glucose, and glycerol at both 30 and 35 °C. These authors report higher volumetric productivity and PHA fractions in CDM, and unaltered specific growth rates for cultivations carried out on glucose or sucrose at 35 °C or 30 °C, respectively. When using glycerol as the carbon source, no biomass formation or significant substrate consumption was observed, probably due to the lack of energy needed to convert the glycerol molecules [50]. As demonstrated by Rodriguez-Contreras et al. who operated a *B. sacchari*-mediated PHB production process at 37 °C, this problem can be overcome by feeding the cells with energy-rich carbohydrates like glucose or sucrose in the first stage (growth phase), and subsequently switching to glycerol feeding in the second phase (PHA accumulation) [39].

Values of 1.29 g/(L·h) (PHB) and 1.87 g/(L·h) (4HB-containing PHA) were achieved for the volumetric PHA productivity in the two conducted bioreactor experiments. These values are considerably higher than that reported for comparable experiments by Rodriguez-Contreras et al., who reported a volumetric productivity of 0.08 g/(L·h) for a two-stage process based on the co-feeding of *B. sacchari* with glucose and glycerol [39], and by Cesário and colleagues, who obtained 0.7 g/(L·h) for fed-batch cultures supplied with glucose and GBL, and 0.5 g/(L·h) when using WSH plus GBL for fed-batch P(3HB-*co*-4HB) production [53]. Here, it has to be emphasized that Cesário et al. [53] used considerably higher GBL dosage than we did in the study at hand; this, on the one hand, resulted in tripling the molar fractions of 4HB in PHA in comparison to our results, but, on the other hand, negatively influenced the overall volumetric PHA productivity as the fundamental economic parameter in PHA production. Regarding the obtained PHA contents in the biomass, our results show final PHA fractions in CDM of 52.4% for PHB, and 71.5% for P(3HB-*co*-4HB), respectively. The results by Cesário and colleagues report 73% PHB in CDM in fed-batch cultures with glucose as the sole carbon source, and 45% P(3HB-*co*-4HB) in CDM with pulse feeding 8 g/L GBL in the accumulation phase followed by continuously feeding GBL at a rate of 2.3 g/h. Fed-batch cultures of *B. sacchari* on WSH plus GBL reported in the same study resulted in a P(3HB-*co*-4HB) fraction in CDM of 27%. Interestingly, the authors found that in *B. sacchari*, the conversion yield of GBL towards 4HB can considerably be improved by supplementing acetate or propionate as additional “stimulants” for the 4HB biosynthesis [53]. Based on the works carried out by Lee et al. with *C. necator*, it was known previously that an increased acetyl-CoA pool from acetate conversion or from propionate ketolysis, respectively, inhibits the conversion of 4HB-CoA to acetyl-CoA, thus preserving a high 4HB-CoA pool available for the P(3HB-*co*-4HB) biosynthesis [67]. Using the mutant strain *B. sacchari* IPT 189, PHA copolyesters consisting of 3HB and 3HV were produced by Rocha et al. by co-feeding sucrose and propionic acid in two-stage bioreactor setups at a volumetric productivity of 1 g/(L·h); in these experiments, the biomass contained a PHA mass fraction of up to 60%, which is higher than in our PHB production process (52.4%), but lower than the value obtained in the present study for P(3HB-*co*-4HB) production (71.5%) [55]. The two-stage co-feeding experiments with *B. sacchari* carried out by Rodriguez-Contreras et al. on glucose and glycerol generated a PHA fraction in CDM that hardly exceeded 10% [39]. Using mixtures of xylose and glucose to mimic differently composed lignocellulosic hydrolysates, Raposo and associates produced PHB by fed-batch cultivations of *B. sacchari* in laboratory bioreactors. Changing the pulse size, feeding rate, and glucose/xylose ratio, the volumetric productivities decreased from 2.7 g/(L·h) (73% PHB in CDM) for pure glucose feeding to 0.07 (11% PHB in CDM) for xylose as the sole carbon source, indicating the inhibitory effect of this pentose sugar [52].

### 4.2. Polymer Characterization:

The obtained data for polymer characterization were in the same range as the results provided by Cesário and colleagues, who extracted PHB and P(3HB-*co*-4HB) from *B. sacchari* biomass, cultivated on WSH, via the same method used in the present study. These authors describe a Mw for PHB of 790 kDa, and between 450 and 590 kDa for P(3HB-*co*-4HB); higher 4HB fractions gradually decreased the Mw values [29]. Our results report a *M*_W_ of 627 kDa for PHB, and 315 kDa for P(3HB-*co*-4HB). The *P_i_* of our sucrose-based polyester samples was higher than the values reported for WSH-based PH. For PHB, we obtained a *P_i_* of 2.66, which is similar to the value obtained for the P(3HB-*co*-4HB) sample (2.51). For comparison, the PHB and P(3HB-*co*-4HB) samples produced by Cesário and colleagues had significantly lower *P_i_*, ranging from 1.4 to 1.7 [29]. Other comparable results were provided by Rosengart et al., who reported a *P_i_* of 2.33 for a *B. sacchari*-based PHB [68]. A considerably lower Mw (200 kDa) was described by Rodriguez-Contreras et al. for PHB obtained by co-feeding *B. sacchari* with glucose and glycerol; in this study, a *P_i_* of 2.5 was reported [39]. Here, it should be noted that glycerol feeding generally results in low molecular mass PHA if compared to sugar-based PHA production, as reported elsewhere [37,68]. This is due to the “endcapping effect”, a phenomenon describing the termination of the *in vivo* PHA chain propagation in the presence of glycerol and other polyols [69]. The melting temperature *T_m_* reported by Cesário and colleagues amounted to 171.7 °C for PHB, and to 158.8 and 164.3 °C for P(3HB-*co*-4HB) with 7.6 or 4.6 mol% of 4HB, respectively [29]. In our case, the *T_m_* for PHB amounted to 177.6 °C, whereas for P(3HB-*co*-4HB) was only 160.9 °C, which matches well with the cited literature data. Our PHB displayed an *X_c_* of 70.9 °C, which is slightly higher than that reported for the WSH-based material (64.8%) [29]. A remarkably low *X_c_* of 24.0% was measured for our P(3HB-*co*-4HB), which is considerably lower than the value reported for P(3HB-*co*-4HB) based on WSH (between 47.2% and 52.3%) [29]. The PHB produced by Rodriguez-Contreras et al. on glucose plus glycerol displayed an *X_c_* of 72.8% and a *T_m_* of 163.3°C [39]. Using PHB-rich biomass from a cultivation of *B. sacchari* on glucose, Rosengart et al. [21] compared the extraction performance of unusual extraction solvents (anisol, phenetole, and cyclohexanone) with the performance of classical chloroform extraction as used in our study, and by Cesário and colleagues [53]. As an outcome, the thermal properties (*T_m_*, *T_g_*, *X_c_*) and molecular mass were fully comparable to the values obtained via chloroform extraction, thus demonstrating the feasibility of switching to sustainable, non-chlorinated alternatives to chloroform [21].

## 5. Conclusions

The highest (up to now) reported productivity for *B. sacchari*-mediated biosynthesis of PHA with building blocks differing from 3HB is described in the present work. Adaptation of the production strain to an elevated temperature optimum of 37 °C makes it a feasible candidate for cost-efficient on-site PHB and P(3HB-*co*-4HB) production starting from cane sugar on the industrial scale. In any case, PHA production facilities should also in future be integrated into the existing production lines for sucrose-based bioethanol production in order to profit from reduced transportation costs, energetic autarky, and in-house availability of extraction solvents for PHA recovery from the biomass. Further efforts should be devoted to high-throughput continuous PHA production by this organism in a chemostat (“chemical environment is static”) process regime. Similar to the results recently obtained by other production strains [70], the application of a multistep-continuous production in a bioreactor cascade displays a viable process-engineering tool to further increase volumetric productivity, and to trigger the distribution of 3HB and 4HB monomers in tailor-made copolyesters. Moreover, the highly effective invertase enzyme excreted by this strain deserves in-depth characterization and might be of interest for applications in food technology. Together with PHA production and other metabolites generated by this strain, such as xylitol or xylonic acid [52], this might open the door to implementing *B. sacchari* as a versatile platform to catalyze a bio-refinery plant starting from inexpensive feedstocks.

## Figures and Tables

**Table 1:** Results of the bioreactor fermentations.

Kinetic Parameter	PHB Production Process (1st Bioreactor Cultivation)	P(3HB-*co*-4HB) Production Process (2nd Bioreactor Cultivation)
*µ_max._* (1/h)	0.41 (*t* = 3.75–6 h)	0.23 (*t* = 6–8 h)
max. CDM (g/L)	70.0 (*t* = 25.25 h)	78.6 (*t* = 32 h)
max. PHA concentration (g/L)	36.8 (*t* = 27.5 h)	55.8 (*t* = 29 h)
max. fraction of PHA in CDM (% *w*/*w*)	53.0 (*t* = 27.5 h)	72.6 (*t* = 29 h)
max. fraction of 4HB in PHA (% mol/mol)	-	1.6 (*t* = 39 h)
Volumetric productivity for PHA (g/L·h)	1.29 (*t* = 0–27.5 h)	1.87 (*t* = 0–39 h)
Yield_CDM/sucorse_ (g/g)	0.18	0.38
Yield 4HB/GBL (g/g)	-	0.05
max. specific productivity *q_P_* (g/(g·h))	0.19 (*t* = 7.25 h)	0.17 (*t* = 17.75 h)
**Material Characterization**		
Weight average molecular mass Mw (kDa)	627 ± 13	315 ± 24
Polydispersity *P_i_* (Mw/Mn)	2.66 ± 0.13	2.51 ± 0.15
Glass transition temperature *T_g_* (°C)	1.0 ± 0.6	1.8 ± 0.2
Melting point *T_m_* (°C)	177.6 ± 0.6	160.9 ± 0.8
Degree of crystallinity *X_c_* (%)	70.9 ± 0.9	24.0 ± 3.6
